# 4-(4-Bromo­phen­yl)-6-(4-chloro­phen­yl)­pyrimidin-2-ylamine

**DOI:** 10.1107/S1600536809002748

**Published:** 2009-01-28

**Authors:** Mujahid Hussain Bukhari, Hamid Latif Siddiqui, Naveed Ahmad, Waseeq Ahmad Siddiqui, Masood Parvez

**Affiliations:** a Institute of Chemistry, University of the Punjab, Lahore 54590, Pakistan; b Department of Chemistry, University of Sargodha, Sargodha, Pakistan; c Department of Chemistry, The University of Calgary, 2500 University Drive NW, Calgary, Alberta, Canada T2N 1N4

## Abstract

The title compound, C_16_H_11_BrClN_3_, contains pairs of mol­ecules lying about inversion centers linked by amino–pyrimidine N—H⋯N hydrogen bonds. The eight-membered rings thus formed are represented by the *R*_2_^2^(8) motif in graph-set notation. The second H atom of the amine group shows a rather weak inter­action with two Br atoms, resulting in bifurcated N—H⋯(Br,Br) hydrogen bonds. The dihedral angles between the mean planes of the benzene rings and the mean plane of the heterocyclic ring are 8.98 (15) and 35.58 (10)°. The Br and Cl atoms show substitutional disorder, with site-occupancy factors of 0.599 (2) and 0.401 (2), respectively.

## Related literature

For related structures, see: Bukhari *et al.* (2008 ▶); Fun *et al.* (2006 ▶); Gallagher *et al.* (2004 ▶). For pharmacological activities of pyrimidines, see: Gangjee *et al.* (1999 ▶); Grivsky *et al.* (1980 ▶); Malik *et al.* (2006 ▶); Rao *et al.* (2003 ▶). For graph-set notation, see: Bernstein *et al.* (1994 ▶).
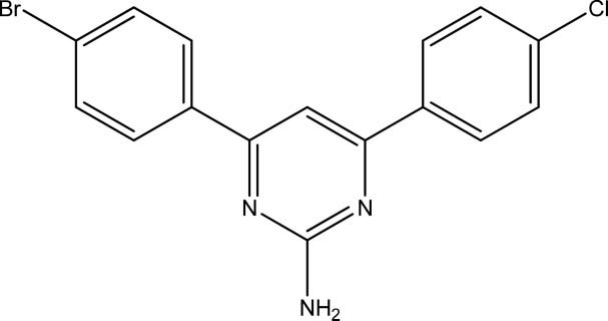


## Experimental

### 

#### Crystal data

C_16_H_11_BrClN_3_
*M* *_r_* = 360.64
Monoclinic, 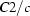

*a* = 39.343 (8) Å
*b* = 3.851 (2) Å
*c* = 22.620 (6) Å
β = 123.81 (2)°
*V* = 2847.6 (18) Å^3^
*Z* = 8
Mo *K*α radiation
μ = 3.07 mm^−1^
*T* = 173 (2) K
0.20 × 0.03 × 0.02 mm

#### Data collection

Nonius KappaCCD diffractometer
Absorption correction: multi-scan (*SORTAV*; Blessing, 1997 ▶) *T*_min_ = 0.579, *T*_max_ = 0.941
8088 measured reflections
2589 independent reflections
1944 reflections with *I* > 2σ(*I*)
*R*_int_ = 0.048

#### Refinement

*R*[*F* ^2^ > 2σ(*F* ^2^)] = 0.039
*wR*(*F* ^2^) = 0.094
*S* = 1.05
2589 reflections
203 parameters
4 restraints
H atoms treated by a mixture of independent and constrained refinement
Δρ_max_ = 0.43 e Å^−3^
Δρ_min_ = −0.58 e Å^−3^

### 

Data collection: *COLLECT* (Hooft, 1998 ▶); cell refinement: *DENZO* (Otwinowski & Minor, 1997 ▶); data reduction: *SCALEPACK* (Otwinowski & Minor, 1997 ▶); program(s) used to solve structure: *SHELXS97* (Sheldrick, 2008 ▶); program(s) used to refine structure: *SHELXL97* (Sheldrick, 2008 ▶); molecular graphics: *ORTEP-3 for Windows* (Farrugia, 1997 ▶); software used to prepare material for publication: *SHELXL97*.

## Supplementary Material

Crystal structure: contains datablocks global, I. DOI: 10.1107/S1600536809002748/fj2180sup1.cif

Structure factors: contains datablocks I. DOI: 10.1107/S1600536809002748/fj2180Isup2.hkl

Additional supplementary materials:  crystallographic information; 3D view; checkCIF report

## Figures and Tables

**Table 1:** Hydrogen-bond geometry (Å, °)

*D*—H⋯*A*	*D*—H	H⋯*A*	*D*⋯*A*	*D*—H⋯*A*
N3—H3*A*⋯Br1^i^	0.92 (4)	2.97 (4)	3.803 (4)	153 (3)
N3—H3*A*⋯Br1^ii^	0.92 (4)	3.11 (4)	3.540 (4)	111 (3)
N3—H3*B*⋯N2^iii^	0.80 (4)	2.28 (5)	3.073 (5)	174 (4)

Symmetry codes: (i) 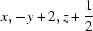; (ii) 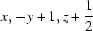; (iii) 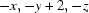.

**Fig. 1.**
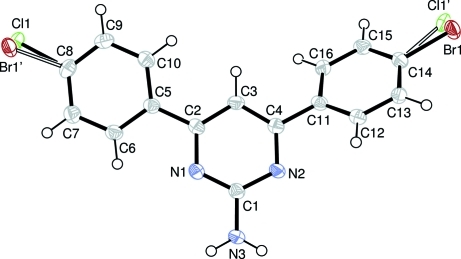
ORTEP-3 (Farrugia, 1997) drawing of (I) with displacement ellipsoids plotted at 50% probability level. Hollow bonds represent smaller fractions of the disordered Br and Cl atoms (Br1 and Cl1, respectively).

**Fig. 2.**
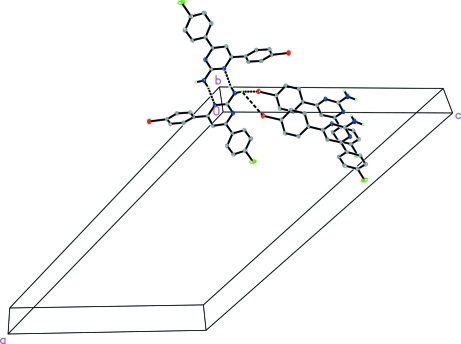
Unit cell packing of (I) showing hydrogen bonds with dashed lines; H-atoms not involved in H-bonds have been omitted.

## supplementary crystallographic information

### Comment

Pyrimidines are a class of biologically active compounds having utility in the pharmaceutical and the agrochemical industries. Compounds with the ring system show pharmacological activity such as antitumor (Gangjee *et al.*, 1999; Grivsky *et al.*, 1980), antiviral (Rao *et al.*, 2003), anti-HIV (Malik *et al.*, 2006), *etc*. In continuation of our research work (Bukhari *et al.*, 2008), we have prepared several pyrimidines. In this article, we report the crystal structure of the title compound, (I).

The structure of (I), (Fig. 1), contains dimeric pairs of molecules lying about inversion centers resulting from N3—H3B···N2^iii^ hydrogen bonds (N3···N2 = 3.071 (5) Å; Table 1 and Fig. 2). The 8-membered rings thus formed represent *R*_2_^2^(8) motif in the graph set notation (Bernstein *et al.*, 1994). The second H-atom of the amine, N3A, shows rather week interactions with two Br atoms representing bifurcated hydrogen bonds (H3A···Br1 2.97 (4) and 3.11 (4) Å). The mean-planes of the two phenyl rings, C5—C10 and C11—C16, are oriented with respect to the mean-plane of the heterocyclic ring at 8.98 (15) and 35.58 (10)°, respectively. The molecular dimensions in (I) agree with the corresponding molecular dimensions reported for 4,6-(diphenyl)pyrimidin-2-amine (Gallagher *et al.*, 2004; Fun *et al.*, 2006). The structure is devoid of any C—H···π(arene) contacts observed in the structures reported above.

### Experimental

The title compound was synthesized by the procedure reported earlier (Bukhari *et al.*, 2008). Crystals of (I) suitable for crystallographic analysis were grown by slow evaporation at 313 K from a solutuion of CHCl_3_ (Yield 58%; m.p. 512–514 K).

### Refinement

The Br and Cl atoms showed substitutional disorder with site occupancy factors refined for Br1 and Cl1 to 0.559 (2) and Br1' and Cl1' to 0.401 (2) values. C—Cl and C—Br distances were constrained using *DFIX* command in *SHELXL97* (Sheldrick, 2008). Though all the H atoms could be distinguished in the difference Fourier map the H-atoms bonded to C-atoms were included at geometrically idealized positions and refined in riding-model approximation with the following constraints: C—H distances were set to 0.95 Å and *U*_iso_(H) = 1.2*U*_eq_(C). H-atoms bonded to N3 were taken from the difference map and were allowed to refine with *U*_iso_ = 1.2 times *U*_eq_ of the parent atom. The final difference map was free of any chemically significant features.

### Crystal data

**Table:**

C_16_H_11_BrClN_3_	*F*(000) = 1440
*M**_r_* = 360.64	*D*_x_ = 1.682 Mg m^−^^3^
Monoclinic, *C*2/*c*	Mo *K*α radiation, λ = 0.71073 Å
Hall symbol: -C 2yc	Cell parameters from 8088 reflections
*a* = 39.343 (8) Å	θ = 3.1–25.3°
*b* = 3.851 (2) Å	µ = 3.07 mm^−^^1^
*c* = 22.620 (6) Å	*T* = 173 K
β = 123.81 (2)°	Needle, colorless
*V* = 2847.6 (18) Å^3^	0.20 × 0.03 × 0.02 mm
*Z* = 8	

### Data collection

**Table:**

Nonius KappaCCD diffractometer	2589 independent reflections
Radiation source: fine-focus sealed tube	1944 reflections with *I* > 2σ(*I*)
graphite	*R*_int_ = 0.048
ω and φ scans	θ_max_ = 25.3°, θ_min_ = 3.1°
Absorption correction: multi-scan (*SORTAV*; Blessing, 1997)	*h* = −46→45
*T*_min_ = 0.579, *T*_max_ = 0.941	*k* = −4→4
8088 measured reflections	*l* = −26→26

### Refinement

**Table:**

Refinement on *F*^2^	Primary atom site location: structure-invariant direct methods
Least-squares matrix: full	Secondary atom site location: difference Fourier map
*R*[*F*^2^ > 2σ(*F*^2^)] = 0.039	Hydrogen site location: inferred from neighbouring sites
*wR*(*F*^2^) = 0.094	H atoms treated by a mixture of independent and constrained refinement
*S* = 1.05	*w* = 1/[σ^2^(*F*_o_^2^) + (0.0319*P*)^2^ + 9.7534*P*] where *P* = (*F*_o_^2^ + 2*F*_c_^2^)/3
2589 reflections	(Δ/σ)_max_ < 0.001
203 parameters	Δρ_max_ = 0.43 e Å^−^^3^
4 restraints	Δρ_min_ = −0.58 e Å^−^^3^

### Special details

**Table:**

Geometry. All e.s.d.'s (except the e.s.d. in the dihedral angle between two l.s. planes) are estimated using the full covariance matrix. The cell e.s.d.'s are taken into account individually in the estimation of e.s.d.'s in distances, angles and torsion angles; correlations between e.s.d.'s in cell parameters are only used when they are defined by crystal symmetry. An approximate (isotropic) treatment of cell e.s.d.'s is used for estimating e.s.d.'s involving l.s. planes.
Refinement. Refinement of *F*^2^ against ALL reflections. The weighted *R*-factor *wR* and goodness of fit *S* are based on *F*^2^, conventional *R*-factors *R* are based on *F*, with *F* set to zero for negative *F*^2^. The threshold expression of *F*^2^ > σ(*F*^2^) is used only for calculating *R*-factors(gt) *etc*. and is not relevant to the choice of reflections for refinement. *R*-factors based on *F*^2^ are statistically about twice as large as those based on *F*, and *R*- factors based on ALL data will be even larger.

### Fractional atomic coordinates and isotropic or equivalent isotropic displacement parameters (Å^2^)

**Table:**

	*x*	*y*	*z*	*U*_iso_*/*U*_eq_	Occ. (<1)
Br1	0.07275 (3)	0.3788 (4)	−0.23596 (6)	0.0284 (2)	0.599 (2)
Cl1	0.28850 (8)	0.8754 (7)	0.45607 (13)	0.0257 (5)	0.599 (2)
Br1'	0.28731 (6)	0.8681 (5)	0.47100 (9)	0.0284 (2)	0.401 (2)
Cl1'	0.08059 (14)	0.3789 (17)	−0.2222 (2)	0.0257 (5)	0.401 (2)
N1	0.09743 (9)	0.9631 (7)	0.16544 (15)	0.0239 (6)	
N2	0.05683 (9)	0.8645 (8)	0.03909 (15)	0.0231 (6)	
N3	0.02841 (10)	1.0562 (9)	0.09907 (18)	0.0302 (8)	
H3A	0.0318 (12)	1.151 (11)	0.139 (2)	0.036*	
H3B	0.0070 (14)	1.073 (11)	0.061 (2)	0.036*	
C1	0.06220 (11)	0.9592 (9)	0.10129 (18)	0.0239 (8)	
C2	0.13100 (11)	0.8621 (9)	0.16862 (18)	0.0223 (7)	
C3	0.12903 (11)	0.7667 (9)	0.10744 (18)	0.0249 (8)	
H3	0.1529	0.6995	0.1095	0.030*	
C4	0.09094 (11)	0.7729 (9)	0.04318 (18)	0.0223 (8)	
C5	0.16972 (10)	0.8644 (9)	0.24074 (18)	0.0233 (7)	
C6	0.17017 (11)	1.0069 (9)	0.29802 (19)	0.0261 (8)	
H6	0.1458	1.1041	0.2901	0.031*	
C7	0.20533 (11)	1.0093 (10)	0.3658 (2)	0.0291 (8)	
H7	0.2053	1.1065	0.4044	0.035*	
C8	0.24065 (9)	0.8673 (10)	0.37647 (15)	0.0283 (8)	
C9	0.24130 (11)	0.7212 (10)	0.3213 (2)	0.0294 (8)	
H9	0.2658	0.6224	0.3297	0.035*	
C10	0.20581 (11)	0.7210 (9)	0.25364 (19)	0.0267 (8)	
H10	0.2060	0.6216	0.2154	0.032*	
C11	0.08642 (11)	0.6800 (9)	−0.02477 (18)	0.0233 (8)	
C12	0.05169 (11)	0.5079 (9)	−0.07802 (19)	0.0244 (8)	
H12	0.0308	0.4484	−0.0708	0.029*	
C13	0.04712 (11)	0.4215 (9)	−0.14167 (18)	0.0245 (8)	
H13	0.0233	0.3047	−0.1782	0.029*	
C14	0.07789 (11)	0.5087 (9)	−0.15093 (16)	0.0239 (8)	
C15	0.11279 (11)	0.6792 (9)	−0.09883 (19)	0.0270 (8)	
H15	0.1336	0.7372	−0.1062	0.032*	
C16	0.11689 (11)	0.7644 (9)	−0.03569 (19)	0.0255 (8)	
H16	0.1408	0.8818	0.0006	0.031*	

### Atomic displacement parameters (Å^2^)

**Table:**

	*U*^11^	*U*^22^	*U*^33^	*U*^12^	*U*^13^	*U*^23^
Br1	0.0312 (5)	0.0367 (4)	0.0141 (5)	0.0009 (4)	0.0106 (4)	−0.0020 (4)
Cl1	0.0220 (8)	0.0441 (11)	0.0050 (9)	0.0017 (7)	0.0039 (7)	−0.0014 (7)
Br1'	0.0312 (5)	0.0367 (4)	0.0141 (5)	0.0009 (4)	0.0106 (4)	−0.0020 (4)
Cl1'	0.0220 (8)	0.0441 (11)	0.0050 (9)	0.0017 (7)	0.0039 (7)	−0.0014 (7)
N1	0.0256 (16)	0.0279 (16)	0.0216 (14)	0.0007 (13)	0.0152 (13)	0.0007 (12)
N2	0.0241 (15)	0.0273 (15)	0.0228 (14)	0.0024 (13)	0.0161 (13)	0.0015 (13)
N3	0.0247 (16)	0.045 (2)	0.0243 (16)	0.0089 (16)	0.0157 (14)	0.0010 (16)
C1	0.0259 (18)	0.0262 (19)	0.0234 (18)	0.0024 (15)	0.0161 (16)	0.0030 (15)
C2	0.0280 (19)	0.0195 (17)	0.0249 (18)	0.0003 (15)	0.0181 (16)	0.0022 (15)
C3	0.0226 (18)	0.0301 (19)	0.0265 (19)	0.0036 (15)	0.0164 (17)	0.0014 (15)
C4	0.0260 (19)	0.0199 (17)	0.0246 (18)	0.0010 (14)	0.0163 (17)	0.0034 (14)
C5	0.0240 (18)	0.0244 (18)	0.0250 (18)	0.0002 (16)	0.0159 (16)	0.0019 (15)
C6	0.0246 (19)	0.0292 (19)	0.0298 (19)	0.0010 (15)	0.0184 (17)	0.0011 (16)
C7	0.029 (2)	0.030 (2)	0.0274 (19)	−0.0011 (16)	0.0154 (17)	−0.0020 (16)
C8	0.0229 (19)	0.0275 (19)	0.0270 (19)	−0.0033 (16)	0.0092 (16)	0.0027 (16)
C9	0.0251 (19)	0.030 (2)	0.035 (2)	0.0061 (16)	0.0181 (18)	0.0052 (16)
C10	0.029 (2)	0.029 (2)	0.0276 (19)	−0.0001 (16)	0.0193 (18)	0.0004 (15)
C11	0.0275 (19)	0.0218 (19)	0.0251 (18)	0.0054 (15)	0.0173 (16)	0.0033 (14)
C12	0.0262 (19)	0.0240 (18)	0.0267 (18)	0.0029 (15)	0.0169 (17)	0.0010 (15)
C13	0.0237 (18)	0.0240 (19)	0.0229 (17)	0.0021 (15)	0.0112 (16)	−0.0009 (15)
C14	0.032 (2)	0.0217 (17)	0.0219 (17)	0.0064 (15)	0.0171 (17)	0.0043 (14)
C15	0.0261 (19)	0.033 (2)	0.0278 (19)	0.0024 (16)	0.0190 (17)	0.0043 (16)
C16	0.0233 (18)	0.030 (2)	0.0228 (18)	0.0000 (16)	0.0126 (16)	−0.0004 (15)

### Geometric parameters (Å, °)

**Table:**

Br1—C14	1.886 (3)	C6—C7	1.380 (5)
Cl1—C8	1.736 (3)	C6—H6	0.9500
Br1'—C8	1.891 (3)	C7—C8	1.384 (5)
Cl1'—C14	1.746 (4)	C7—H7	0.9500
N1—C1	1.337 (5)	C8—C9	1.382 (5)
N1—C2	1.340 (4)	C9—C10	1.384 (5)
N2—C4	1.340 (4)	C9—H9	0.9500
N2—C1	1.352 (4)	C10—H10	0.9500
N3—C1	1.354 (4)	C11—C12	1.388 (5)
N3—H3A	0.92 (4)	C11—C16	1.391 (5)
N3—H3B	0.80 (4)	C12—C13	1.388 (5)
C2—C3	1.392 (5)	C12—H12	0.9500
C2—C5	1.489 (5)	C13—C14	1.380 (5)
C3—C4	1.391 (5)	C13—H13	0.9500
C3—H3	0.9500	C14—C15	1.380 (5)
C4—C11	1.489 (5)	C15—C16	1.385 (5)
C5—C10	1.395 (5)	C15—H15	0.9500
C5—C6	1.398 (5)	C16—H16	0.9500
			
C1—N1—C2	116.8 (3)	C9—C8—Br1'	121.8 (3)
C4—N2—C1	115.4 (3)	C7—C8—Br1'	116.4 (3)
C1—N3—H3A	118 (3)	C10—C9—C8	119.0 (3)
C1—N3—H3B	119 (3)	C10—C9—H9	120.5
H3A—N3—H3B	121 (4)	C8—C9—H9	120.5
N1—C1—N2	126.8 (3)	C9—C10—C5	121.0 (3)
N1—C1—N3	116.1 (3)	C9—C10—H10	119.5
N2—C1—N3	117.0 (3)	C5—C10—H10	119.5
N1—C2—C3	121.0 (3)	C12—C11—C16	119.2 (3)
N1—C2—C5	115.7 (3)	C12—C11—C4	120.3 (3)
C3—C2—C5	123.2 (3)	C16—C11—C4	120.5 (3)
C4—C3—C2	117.8 (3)	C11—C12—C13	120.8 (3)
C4—C3—H3	121.1	C11—C12—H12	119.6
C2—C3—H3	121.1	C13—C12—H12	119.6
N2—C4—C3	122.1 (3)	C14—C13—C12	118.7 (3)
N2—C4—C11	116.9 (3)	C14—C13—H13	120.6
C3—C4—C11	121.0 (3)	C12—C13—H13	120.6
C10—C5—C6	118.4 (3)	C13—C14—C15	121.8 (3)
C10—C5—C2	121.9 (3)	C13—C14—Cl1'	125.4 (3)
C6—C5—C2	119.7 (3)	C15—C14—Cl1'	112.4 (3)
C7—C6—C5	121.4 (3)	C13—C14—Br1	119.0 (3)
C7—C6—H6	119.3	C15—C14—Br1	119.2 (3)
C5—C6—H6	119.3	C14—C15—C16	118.9 (3)
C6—C7—C8	118.6 (3)	C14—C15—H15	120.6
C6—C7—H7	120.7	C16—C15—H15	120.6
C8—C7—H7	120.7	C15—C16—C11	120.7 (3)
C9—C8—C7	121.7 (3)	C15—C16—H16	119.6
C9—C8—Cl1	112.9 (3)	C11—C16—H16	119.6
C7—C8—Cl1	125.3 (3)		
			
C2—N1—C1—N2	−0.1 (5)	C7—C8—C9—C10	0.8 (6)
C2—N1—C1—N3	178.9 (3)	Cl1—C8—C9—C10	−175.4 (3)
C4—N2—C1—N1	−1.5 (5)	Br1'—C8—C9—C10	178.3 (3)
C4—N2—C1—N3	179.5 (3)	C8—C9—C10—C5	−0.2 (6)
C1—N1—C2—C3	1.5 (5)	C6—C5—C10—C9	−0.4 (5)
C1—N1—C2—C5	−179.2 (3)	C2—C5—C10—C9	−179.0 (3)
N1—C2—C3—C4	−1.3 (5)	N2—C4—C11—C12	−35.5 (5)
C5—C2—C3—C4	179.5 (3)	C3—C4—C11—C12	145.1 (4)
C1—N2—C4—C3	1.7 (5)	N2—C4—C11—C16	144.5 (3)
C1—N2—C4—C11	−177.7 (3)	C3—C4—C11—C16	−34.9 (5)
C2—C3—C4—N2	−0.4 (5)	C16—C11—C12—C13	−0.3 (5)
C2—C3—C4—C11	179.0 (3)	C4—C11—C12—C13	179.7 (3)
N1—C2—C5—C10	170.9 (3)	C11—C12—C13—C14	0.3 (5)
C3—C2—C5—C10	−9.9 (5)	C12—C13—C14—C15	−0.2 (5)
N1—C2—C5—C6	−7.7 (5)	C12—C13—C14—Cl1'	172.1 (4)
C3—C2—C5—C6	171.5 (3)	C12—C13—C14—Br1	177.9 (3)
C10—C5—C6—C7	0.4 (5)	C13—C14—C15—C16	0.0 (5)
C2—C5—C6—C7	179.1 (3)	Cl1'—C14—C15—C16	−173.1 (4)
C5—C6—C7—C8	0.1 (5)	Br1—C14—C15—C16	−178.1 (3)
C6—C7—C8—C9	−0.7 (6)	C14—C15—C16—C11	0.0 (5)
C6—C7—C8—Cl1	174.9 (3)	C12—C11—C16—C15	0.1 (5)
C6—C7—C8—Br1'	−178.4 (3)	C4—C11—C16—C15	−179.9 (3)

### Hydrogen-bond geometry (Å, °)

**Table:**

*D*—H···*A*	*D*—H	H···*A*	*D*···*A*	*D*—H···*A*
N3—H3A···Br1^i^	0.92 (4)	2.97 (4)	3.803 (4)	153 (3)
N3—H3A···Br1^ii^	0.92 (4)	3.11 (4)	3.540 (4)	111 (3)
N3—H3B···N2^iii^	0.80 (4)	2.28 (5)	3.073 (5)	174 (4)

Symmetry codes: (i) *x*, −*y*+2, *z*+1/2; (ii) *x*, −*y*+1, *z*+1/2; (iii) −*x*, −*y*+2, −*z*.
